# On Lyapunov-type inequalities for $(p,q)$-Laplacian systems

**DOI:** 10.1186/s13660-017-1377-0

**Published:** 2017-05-04

**Authors:** Mohamed Jleli, Bessem Samet

**Affiliations:** Department of Mathematics, College of Science, King Saud University, P.O. Box 2455, Riyadh, 11451 Saudi Arabia

**Keywords:** 26D10, 15A42, 39B72, Lyapunov-type inequalities, $(p,q)$-Laplacian, system, generalized eigenvalues, generalized spectrum

## Abstract

We establish Lyapunov-type inequalities for a system involving one-dimensional $(p_{i},q_{i})$-Laplacian operators ($i=1,2$). Next, the obtained inequalities are used to derive some geometric properties of the generalized spectrum associated to the considered problem.

## Introduction

In this paper, we are concerned with the following system involving one-dimensional $(p_{i},q_{i})$-Laplacian operators ($i=1,2$):

$$\begin{aligned} \mbox{(S)}:\quad \textstyle\begin{cases} -( \vert u'(x) \vert ^{p_{1}-2}u'(x))'-( \vert u'(x) \vert ^{q_{1}-2}u'(x))'=f(x) \vert u(x) \vert ^{\alpha-2} \vert v(x) \vert ^{\beta}u(x),\\ -( \vert v'(x) \vert ^{p_{2}-2}v'(x))'-( \vert v'(x) \vert ^{q_{2}-2}v'(x))'=g(x) \vert u(x) \vert ^{\alpha} \vert v(x) \vert ^{\beta-2}v(x) \end{cases}\displaystyle \end{aligned}$$

on the interval $(a,b)$, under Dirichlet boundary conditions

$$\mbox{(DBC)}:\quad u(a)=u(b)=v(a)=v(b)=0. $$

System (S) is investigated under the assumptions

$$\alpha\geq 2,\qquad \beta\geq 2,\qquad p_{i}\geq 2,\qquad q_{i}\geq 2, \quad i=1,2, $$

and

1 $$ \frac{2\alpha}{p_{1}+q_{1}}+\frac{2\beta}{p_{2}+q_{2}}=1. $$

We suppose also that *f* and *g* are two nonnegative real-valued functions such that $(f,g)\in L^{1}(a,b)\times L^{1}(a,b)$. We establish a Lyapunov-type inequality for problem (S)-(DBC). Next, we use the obtained inequality to derive some geometric properties of the generalized spectrum associated to the considered problem.

The standard Lyapunov inequality [1] (see also [2]) states that if the boundary value problem

$$\begin{aligned} \textstyle\begin{cases} u''(t)+q(t)u(t)=0,\quad a< t< b,\\ u(a)=u(b)=0, \end{cases}\displaystyle \end{aligned}$$

has a nontrivial solution, where $q: [a,b]\to \mathbb{R}$ is a continuous function, then

2 $$ \int_{a}^{b} \bigl\vert q(t) \bigr\vert \,dt> \frac{4}{b-a}. $$

Inequality (2) was successfully applied to oscillation theory, stability criteria for periodic differential equations, estimates for intervals of disconjugacy, and eigenvalue bounds for ordinary differential equations. In [3] (see also [4]), Elbert extended inequality (2) to the one-dimensional *p*-Laplacian equation. More precisely, he proved that, if *u* is a nontrivial solution of the problem

$$\begin{aligned} \textstyle\begin{cases} ( \vert u' \vert ^{p-2}u')'+h(t) \vert u \vert ^{p-2}u=0,\quad a< t< b,\\ u(a)=u(b)=0, \end{cases}\displaystyle \end{aligned}$$

where $1< p<\infty$ and $h\in L^{1}(a,b)$, then

3 $$ \int_{a}^{b} \bigl\vert h(t) \bigr\vert \,dt> \frac{2^{p}}{(b-a)^{p-1}}. $$

Observe that for $p=2$, (3) reduces to (2). Inequality (3) was extended in [5] to the following problem involving the *φ*-Laplacian operator:

$$\begin{aligned} \textstyle\begin{cases} (\varphi(u'))'+w(t)\varphi(u)=0,\quad a< t< b,\\ u(a)=u(b)=0, \end{cases}\displaystyle \end{aligned}$$

where $\varphi: \mathbb{R}\to \mathbb{R}$ is a convex nondecreasing function satisfying a $\Delta_{2}$ condition. In [6], Nápoli and Pinasco considered the quasilinear system of resonant type

4 $$\begin{aligned} \textstyle\begin{cases} -( \vert u'(x) \vert ^{p-2}u'(x))'= f(x) \vert u(x) \vert ^{\alpha-2} \vert v(x) \vert ^{\beta}u(x),\\ -( \vert v'(x) \vert ^{q-2}v'(x))'= g(x) \vert u(x) \vert ^{\alpha} \vert v(x) \vert ^{\beta-2}v(x) \end{cases}\displaystyle \end{aligned}$$

on the interval $(a,b)$, with Dirichlet boundary conditions

5 $$ u(a)=u(b)=v(a)=v(b)=0. $$

Under the assumptions $p,q>1$, $f,g\in L^{1}(a,b)$, $f,g\geq 0$, $\alpha,\beta\geq 0$, and

$$\frac{\alpha}{p}+\frac{\beta}{q}=1, $$

it was proved (see [6], Theorem 1.5) that if (4)-(5) has a nontrivial solution, then

6 $$ 2^{\alpha+\beta} \leq (b-a)^{\frac{\alpha}{p'}+\frac{\beta}{q'}} \biggl( \int_{a}^{b} f(x)\,dx \biggr)^{\frac{\alpha}{p}} \biggl( \int_{a}^{b} g(x)\,dx \biggr)^{\frac{\beta}{q}}, $$

where $p'=\frac{p}{p-1}$ and $q'=\frac{q}{q-1}$. Some nice applications to generalized eigenvalues are also presented in [6]. Different generalizations and extensions of inequality (6) were obtained by many authors. In this direction, we refer the reader to [7–16] and the references therein. For other results concerning Lyapunov-type inequalities, we refer the reader to [17–29] and the references therein.

## Lyapunov-type inequalities

A Lyapunov-type inequality for problem (S)-(DBC) is established in this section, and some particular cases are discussed.

### Theorem 2.1

*If* (S)-(DBC) *admits a nontrivial solution* $(u,v)\in C^{2}[a,b]\times C^{2}[a,b]$, *then*

7 $$\begin{aligned} & \biggl[\min \biggl\{ \frac{2^{p_{1}}}{(b-a)^{p_{1}-1}},\frac{2^{q_{1}}}{(b-a)^{q_{1}-1}} \biggr\} \biggr]^{\frac{2\alpha}{p_{1}+q_{1}}} \biggl[\min \biggl\{ \frac{2^{p_{2}}}{(b-a)^{p_{2}-1}}, \frac{2^{q_{2}}}{(b-a)^{q_{2}-1}} \biggr\} \biggr]^{\frac{2\beta}{p_{2}+q_{2}}} \\ &\quad \leq \biggl(\frac{1}{2} \int_{a}^{b} f(x)\,dx \biggr)^{\frac{2\alpha}{p_{1}+q_{1}}} \biggl( \frac{1}{2} \int_{a}^{b} g(x)\,dx \biggr)^{\frac{2\beta}{p_{2}+q_{2}}}. \end{aligned}$$

### Proof

Let $(u,v)\in C^{2}[a,b]\times C^{2}[a,b]$ be a nontrivial solution to (S)-(DBC). Let $(x_{0},y_{0})\in (a,b)\times (a,b)$ be such that

$$\bigl\vert u(x_{0}) \bigr\vert =\max\bigl\{ \bigl\vert u(x) \bigr\vert :\, a\leq x\leq b\bigr\} $$

and

$$\bigl\vert v(y_{0}) \bigr\vert =\max\bigl\{ \bigl\vert v(x) \bigr\vert :\, a\leq x\leq b\bigr\} . $$

From the boundary conditions (DBC), we can write that

$$2u(x_{0})= \int_{a}^{x_{0}} u'(x)\,dx - \int_{x_{0}}^{b} u'(x)\,dx, $$

which yields

$$2 \bigl\vert u(x_{0}) \bigr\vert \leq \int_{a}^{b} \bigl\vert u'(x) \bigr\vert \,dx. $$

Using Hölder’s inequality with parameters $p_{1}$ and $p_{1}'=\frac{p_{1}}{p_{1}-1}$, we get

$$2 \bigl\vert u(x_{0}) \bigr\vert \leq (b-a)^{\frac{1}{p_{1}'}} \biggl( \int_{a}^{b} \bigl\vert u'(x) \bigr\vert ^{p_{1}}\,dx \biggr)^{\frac{1}{p_{1}}}, $$

that is,

8 $$ \frac{2^{p_{1}}}{(b-a)^{p_{1}-1}} \bigl\vert u(x_{0}) \bigr\vert ^{p_{1}} \leq \int_{a}^{b} \bigl\vert u'(x) \bigr\vert ^{p_{1}}\,dx. $$

Similarly, using Hölder’s inequality with parameters $q_{1}$ and $q_{1}'=\frac{q_{1}}{q_{1}-1}$, we get

9 $$ \frac{2^{q_{1}}}{(b-a)^{q_{1}-1}} \bigl\vert u(x_{0}) \bigr\vert ^{q_{1}} \leq \int_{a}^{b} \bigl\vert u'(x) \bigr\vert ^{q_{1}}\,dx. $$

By repeating the same argument for the function *v*, we obtain

10 $$ \frac{2^{p_{2}}}{(b-a)^{p_{2}-1}} \bigl\vert v(y_{0}) \bigr\vert ^{p_{2}} \leq \int_{a}^{b} \bigl\vert v'(x) \bigr\vert ^{p_{2}}\,dx $$

and

11 $$ \frac{2^{q_{2}}}{(b-a)^{q_{2}-1}} \bigl\vert v(y_{0}) \bigr\vert ^{q_{2}} \leq \int_{a}^{b} \bigl\vert v'(x) \bigr\vert ^{q_{2}}\,dx. $$

Now, multiplying the first equation of (S) by *u* and integrating over $(a,b)$, we obtain

12 $$ \int_{a}^{b} \bigl\vert u'(x) \bigr\vert ^{p_{1}}\,dx+ \int_{a}^{b} \bigl\vert u'(x) \bigr\vert ^{q_{1}}\,dx= \int_{a}^{b} f(x) \bigl\vert u(x) \bigr\vert ^{\alpha}\bigl\vert v(x) \bigr\vert ^{\beta}\,dx. $$

Multiplying the second equation of (S) by *v* and integrating over $(a,b)$, we obtain

13 $$ \int_{a}^{b} \bigl\vert v'(x) \bigr\vert ^{p_{2}}\,dx+ \int_{a}^{b} \bigl\vert v'(x) \bigr\vert ^{q_{2}}\,dx= \int_{a}^{b} g(x) \bigl\vert u(x) \bigr\vert ^{\alpha}\bigl\vert v(x) \bigr\vert ^{\beta}\,dx. $$

Using (8), (9) and (12), we obtain

$$\bigl\vert u(x_{0}) \bigr\vert ^{\alpha}\bigl\vert v(y_{0}) \bigr\vert ^{\beta}\int_{a}^{b} f(x) \,dx\geq \frac{2^{p_{1}}}{(b-a)^{p_{1}-1}} \bigl\vert u(x_{0}) \bigr\vert ^{p_{1}}+\frac{2^{q_{1}}}{(b-a)^{q_{1}-1}} \bigl\vert u(x_{0}) \bigr\vert ^{q_{1}}, $$

which yields

$$\bigl\vert u(x_{0}) \bigr\vert ^{\alpha}\bigl\vert v(y_{0}) \bigr\vert ^{\beta}\int_{a}^{b} f(x) \,dx\geq \min \biggl\{ \frac{2^{p_{1}}}{(b-a)^{p_{1}-1}},\frac{2^{q_{1}}}{(b-a)^{q_{1}-1}} \biggr\} \bigl( \bigl\vert u(x_{0}) \bigr\vert ^{p_{1}}+ \bigl\vert u(x_{0}) \bigr\vert ^{q_{1}} \bigr). $$

Using the inequality

$$A+B\geq 2\sqrt{A}\sqrt{B} $$

with $A= \vert u(x_{0}) \vert ^{p_{1}}$ and $B= \vert u(x_{0}) \vert ^{q_{1}}$, we get

14 $$ \min \biggl\{ \frac{2^{p_{1}+1}}{(b-a)^{p_{1}-1}},\frac{2^{q_{1}+1}}{(b-a)^{q_{1}-1}} \biggr\} \leq \bigl\vert u(x_{0}) \bigr\vert ^{\alpha-\frac{p_{1}+q_{1}}{2}} \bigl\vert v(y_{0}) \bigr\vert ^{\beta} \int_{a}^{b} f(x)\,dx. $$

Similarly, using (10), (11) and (13), we obtain

15 $$ \min \biggl\{ \frac{2^{p_{2}+1}}{(b-a)^{p_{2}-1}},\frac{2^{q_{2}+1}}{(b-a)^{q_{2}-1}} \biggr\} \leq \bigl\vert u(x_{0}) \bigr\vert ^{\alpha} \bigl\vert v(y_{0}) \bigr\vert ^{\beta-\frac{p_{2}+q_{2}}{2}} \int_{a}^{b} g(x)\,dx. $$

Raising inequality (14) to a power $e_{1}>0$, inequality (15) to a power $e_{2}>0$, and multiplying the resulting inequalities, we obtain

$$\begin{aligned} & \biggl[\min \biggl\{ \frac{2^{p_{1}+1}}{(b-a)^{p_{1}-1}},\frac{2^{q_{1}+1}}{(b-a)^{q_{1}-1}} \biggr\} \biggr]^{e_{1}} \biggl[\min \biggl\{ \frac{2^{p_{2}+1}}{(b-a)^{p_{2}-1}},\frac{2^{q_{2}+1}}{(b-a)^{q_{2}-1}} \biggr\} \biggr]^{e_{2}} \\ &\quad \leq \bigl\vert u(x_{0}) \bigr\vert ^{ (\alpha-\frac{p_{1}+q_{1}}{2} )e_{1}+\alpha e_{2}} \bigl\vert v(y_{0}) \bigr\vert ^{\beta e_{1}+ (\beta-\frac{p_{2}+q_{2}}{2} )e_{2}} \biggl( \int_{a}^{b} f(x)\,dx \biggr)^{e_{1}} \biggl( \int_{a}^{b} g(x)\,dx \biggr)^{e_{2}}. \end{aligned}$$

Next, we take $(e_{1},e_{2})$ any solution of the homogeneous linear system

$$\begin{aligned} \textstyle\begin{cases} (\alpha-\frac{p_{1}+q_{1}}{2} )e_{1}+\alpha e_{2}= 0,\\ \beta e_{1}+ (\beta-\frac{p_{2}+q_{2}}{2} )e_{2}=0. \end{cases}\displaystyle \end{aligned}$$

Using (1), we may take

$$\begin{aligned} \textstyle\begin{cases} e_{1}= \alpha,\\ e_{2}=\frac{\beta(p_{1}+q_{1})}{p_{2}+q_{2}}. \end{cases}\displaystyle \end{aligned}$$

Therefore, we obtain

$$\begin{aligned} &2^{\alpha+\frac{\beta(p_{1}+q_{1})}{p_{2}+q_{2}}} \biggl[\min \biggl\{ \frac{2^{p_{1}}}{(b-a)^{p_{1}-1}},\frac{2^{q_{1}}}{(b-a)^{q_{1}-1}} \biggr\} \biggr]^{\alpha} \biggl[\min \biggl\{ \frac{2^{p_{2}}}{(b-a)^{p_{2}-1}}, \frac{2^{q_{2}}}{(b-a)^{q_{2}-1}} \biggr\} \biggr]^{\frac{\beta(p_{1}+q_{1})}{p_{2}+q_{2}}} \\ &\quad \leq \biggl( \int_{a}^{b} f(x)\,dx \biggr)^{\alpha} \biggl( \int_{a}^{b} g(x)\,dx \biggr)^{\frac{\beta(p_{1}+q_{1})}{p_{2}+q_{2}}}. \end{aligned}$$

Using again (1), we get

$$\begin{aligned} &2 \biggl[\min \biggl\{ \frac{2^{p_{1}}}{(b-a)^{p_{1}-1}},\frac{2^{q_{1}}}{(b-a)^{q_{1}-1}} \biggr\} \biggr]^{\frac{2\alpha}{p_{1}+q_{1}}} \biggl[\min \biggl\{ \frac{2^{p_{2}}}{(b-a)^{p_{2}-1}},\frac{2^{q_{2}}}{(b-a)^{q_{2}-1}} \biggr\} \biggr]^{\frac{2\beta}{p_{2}+q_{2}}} \\ &\quad \leq \biggl( \int_{a}^{b} f(x)\,dx \biggr)^{\frac{2\alpha}{p_{1}+q_{1}}} \biggl( \int_{a}^{b} g(x)\,dx \biggr)^{\frac{2\beta}{p_{2}+q_{2}}}, \end{aligned}$$

which proves Theorem 2.1. □

As a consequence of Theorem 2.1, we deduce the following result for the case of a single equation.

### Corollary 1

*Let us assume that there exists a nontrivial solution of*

$$\begin{aligned} \textstyle\begin{cases} -( \vert u'(x) \vert ^{p-2}u'(x))'-( \vert u'(x) \vert ^{q-2}u'(x))'=f(x) \vert u(x) \vert ^{\frac{p+q}{2}-2}u(x),\quad x\in (a,b),\\ u(a)=u(b)=0, \end{cases}\displaystyle \end{aligned}$$

*where* $p>1$, $q>1$, $f\geq 0$, *and* $f\in L^{1}(a,b)$. *Then*

$$\min \biggl\{ \frac{2^{p}}{(b-a)^{p-1}},\frac{2^{q}}{(b-a)^{q-1}} \biggr\} \leq \frac{1}{2} \int_{a}^{b} f(x)\,dx. $$

### Proof

An application of Theorem 2.1 with

$$p_{1}=p_{2}=p,\qquad q_{1}=q_{2}=q,\qquad \alpha=\frac{p+q}{2},\qquad \beta=0,\qquad v=u,\qquad g=f, $$

yields the desired result. □

### Remark 1

Taking $f=2h$ and $q=p$ in Corollary 1, we obtain Lyapunov-type inequality (3) for the one-dimensional *p*-Laplacian equation.

### Remark 2

Taking $p_{1}=q_{1}=p$ and $p_{2}=q_{2}=q$ in Theorem 2.1, we obtain Lyapunov-type inequality (6).

## Generalized eigenvalues

The concept of generalized eigenvalues was introduced by Protter [30] for a system of linear elliptic operators. The first work dealing with generalized eigenvalues for *p*-Laplacian systems is due to Nápoli and Pinasco [6]. Inspired by that work, we present in this section some applications to generalized eigenvalues related to problem (S)-(DBC).

Let us consider the generalized eigenvalue problem

$$\begin{aligned} \mbox{(S)}_{\lambda,\mu}:\quad \textstyle\begin{cases} -( \vert u'(x) \vert ^{p_{1}-2}u'(x))'-( \vert u'(x) \vert ^{q_{1}-2}u'(x))'=\lambda \alpha w(x) \vert u(x) \vert ^{\alpha-2} \vert v(x) \vert ^{\beta}u(x),\\ -( \vert v'(x) \vert ^{p_{2}-2}v'(x))'-( \vert v'(x) \vert ^{q_{2}-2}v'(x))'=\mu \beta w(x) \vert u(x) \vert ^{\alpha} \vert v(x) \vert ^{\beta-2}v(x), \end{cases}\displaystyle \end{aligned}$$

on the interval $(a,b)$, with Dirichlet boundary conditions (DBC). If problem $\mbox{(S)}_{\lambda,\mu}$-(DBC) admits a nontrivial solution $(u,v)\in C^{2}[a,b]\times C^{2}[a,b]$, we say that $(\lambda,\mu)$ is a generalized eigenvalue of $\mbox{(S)}_{\lambda,\mu}$-(DBC). The set of generalized eigenvalues is called generalized spectrum, and it is denoted by *σ*.

We assume that

$$\alpha\geq 2,\qquad \beta\geq 2,\qquad p_{i}\geq 2,\qquad q_{i}\geq 2, \quad i=1,2, \qquad w\geq 0,\qquad w\in L^{1}(a,b), $$

and (1) is satisfied.

The following result provides lower bounds of the generalized eigenvalues of $\mbox{(S)}_{\lambda,\mu}$-(DBC).

### Theorem 3.1

*Let* $(\lambda,\mu)$ *be a generalized eigenvalue of* $\mathrm{(S)}_{\lambda,\mu}$-(DBC). *Then*

16 $$ \mu\geq h(\lambda), $$

*where* $h: (0,\infty)\to (0,\infty)$ *is the function defined by*

$$h(t)=\frac{1}{\beta} \biggl(\frac{C}{t^{\frac{2\alpha}{p_{1}+q_{1}}}\int_{a}^{b} w(x)\,dx} \biggr)^{\frac{p_{2}+q_{2}}{2\beta}},\quad t>0, $$

*with*

$$\begin{aligned} \alpha^{\frac{2\alpha}{p_{1}+q_{1}}} C =&2 \biggl[\min \biggl\{ \frac{2^{p_{1}}}{(b-a)^{p_{1}-1}}, \frac{2^{q_{1}}}{(b-a)^{q_{1}-1}} \biggr\} \biggr]^{\frac{2\alpha}{p_{1}+q_{1}}} \\ &{}\times\biggl[\min \biggl\{ \frac{2^{p_{2}}}{(b-a)^{p_{2}-1}},\frac{2^{q_{2}}}{(b-a)^{q_{2}-1}} \biggr\} \biggr]^{\frac{2\beta}{p_{2}+q_{2}}}. \end{aligned}$$

### Proof

Let $(\lambda,\mu)$ be a generalized eigenpair, and let $u,v$ be the corresponding nontrivial solutions. By replacing in Lyapunov-type inequality (7) the functions

$$f(x)=\alpha \lambda w(x),\qquad g(x)=\beta \mu w(x), $$

and using condition (1), we obtain

$$2M\leq \alpha^{\frac{2\alpha}{p_{1}+q_{1}}} \lambda^{\frac{2\alpha}{p_{1}+q_{1}}}\beta^{\frac{2\beta}{p_{2}+q_{2}}} \mu^{\frac{2\beta}{p_{2}+q_{2}}} \int_{a}^{b} w(x)\,dx, $$

where

$$M= \biggl[\min \biggl\{ \frac{2^{p_{1}}}{(b-a)^{p_{1}-1}},\frac{2^{q_{1}}}{(b-a)^{q_{1}-1}} \biggr\} \biggr]^{\frac{2\alpha}{p_{1}+q_{1}}} \biggl[\min \biggl\{ \frac{2^{p_{2}}}{(b-a)^{p_{2}-1}},\frac{2^{q_{2}}}{(b-a)^{q_{2}-1}} \biggr\} \biggr]^{\frac{2\beta}{p_{2}+q_{2}}}. $$

Hence, we have

$$\mu^{\frac{2\beta}{p_{2}+q_{2}}}\geq \frac{C}{\lambda^{\frac{2\alpha}{p_{1}+q_{1}}}\beta^{\frac{2\beta}{p_{2}+q_{2}}}\int_{a}^{b} w(x)\,dx}, $$

which yields

$$\mu\geq \frac{1}{\beta} \biggl(\frac{C}{\lambda^{\frac{2\alpha}{p_{1}+q_{1}}}\int_{a}^{b} w(x)\,dx} \biggr)^{\frac{p_{2}+q_{2}}{2\beta}}, $$

and the proof is finished. □

As consequences of the previous obtained result, we deduce the following Protter’s type results for the generalized spectrum.

### Corollary 2

*There exists a constant* $c_{a,b}>0$ *that depends on* *a* *and* *b* *such that no point of the generalized spectrum* *σ* *is contained in the ball* $B(0,c_{a,b})$, *where*

$$B(0,c_{a,b})= \bigl\{ x=(x_{1},x_{2})\in \mathbb{R}^{2}:\, \Vert x \Vert _{\infty}< c_{a,b} \bigr\} , $$

*and* $\Vert \cdot \Vert _{\infty}$ *is the Chebyshev norm in* $\mathbb{R}^{2}$.

### Proof

Let $(\lambda,\mu)\in \sigma$. From (16), we obtain easily that

17 $$ \lambda^{\frac{2\alpha}{p_{1}+q_{1}}} \mu^{\frac{2\beta}{p_{2}+q_{2}}}\geq \frac{C}{\beta^{\frac{2\beta}{p_{2}+q_{2}}}\int_{a}^{b} w(x)\,dx}. $$

On the other hand, using condition (1), we have

$$\lambda^{\frac{2\alpha}{p_{1}+q_{1}}} \mu^{\frac{2\beta}{p_{2}+q_{2}}}\leq \bigl\Vert (\lambda,\mu) \bigr\Vert _{\infty}^{\frac{2\alpha}{p_{1}+q_{1}}+\frac{2\beta}{p_{2}+q_{2}}}= \bigl\Vert (\lambda,\mu) \bigr\Vert _{\infty}. $$

Therefore, we obtain

$$\bigl\Vert (\lambda,\mu) \bigr\Vert _{\infty}\geq c_{a,b}, $$

where

$$c_{a,b}=\frac{C}{\beta^{\frac{2\beta}{p_{2}+q_{2}}}\int_{a}^{b} w(x)\,dx}. $$

The proof is finished. □

### Corollary 3

*Let* $(\lambda,\mu)$ *be fixed*. *There exists an interval* *J* *of sufficiently small measure such that*, *if* $I=[a,b]\subset J$, *then there are no nontrivial solutions of* $\mathrm{(S)}_{\lambda,\mu}$-(DBC).

### Proof

Suppose that $\mbox{(S)}_{\lambda,\mu}$-(DBC) admits a nontrivial solution. Since $C\to +\infty$ as $b-a\to 0^{+}$, where *C* is defined in Theorem 3.1, there exists $\delta>0$ such that

$$b-a< \delta \quad \implies\quad \frac{C}{\int_{a}^{b} w(x)\,dx}>\lambda^{\frac{2\alpha}{p_{1}+q_{1}}} \mu^{\frac{2\beta}{p_{2}+q_{2}}} \beta^{\frac{2\beta}{p_{2}+q_{2}}}. $$

Let $J=[a,a+\delta]$. Hence, if $I\subset J$, we have

$$\frac{C}{\beta^{\frac{2\beta}{p_{2}+q_{2}}} \int_{a}^{b} w(x)\,dx}>\lambda^{\frac{2\alpha}{p_{1}+q_{1}}} \mu^{\frac{2\beta}{p_{2}+q_{2}}}, $$

which is a contradiction with (17). Therefore, if $I\subset J$, there are no nontrivial solutions of $\mbox{(S)}_{\lambda,\mu}$-(DBC). □

## Conclusion

Lyapunov-type inequalities for a system of differential equations involving one-dimensional $(p_{i},q_{i})$-Laplacian operators ($i=1,2$) are derived. It was shown that such inequalities are very useful to obtain geometric characterizations of the generalized spectrum associated to the considered problem.
